# Addressing Reproducibility of MXene Synthesis Using Electrochemical and Chemical Treatment of Ti_3_AlC_2_ With Tetramethylammonium Hydroxide

**DOI:** 10.1002/smtd.70878

**Published:** 2026-07-28

**Authors:** Bartosz Gurzęda, Nicolas Boulanger, Mads Ry Vogel Jørgensen, Innokenty Kantor, Christoph Hennig, Alexandr V. Talyzin

**Affiliations:** ^1^ Department of Physics Umeå University Umeå Sweden; ^2^ Department of Chemistry and iNANO Aarhus University Aarhus Denmark; ^3^ MAX IV Laboratory Lund University Lund Sweden; ^4^ Institute of Resource Ecology Helmholtz‐Zentrum Dresden‐Rossendorf Dresden Germany; ^5^ The Rossendorf Beamline at ESRF The European Synchrotron Grenoble France

**Keywords:** in situ, MXene, reproducibility, synthesis, XRD

## Abstract

Electrochemical etching of Ti_3_AlC_2_ in an aqueous electrolyte consisting of 1 M NH_4_Cl and 0.2 M tetramethylammonium hydroxide (TMAOH) is widely cited as a method to produce fluorine‐free MXene. However, an alternative method for producing MXene by purely chemical etching of Ti_3_AlC_2_ with an aqueous TMAOH has also been reported. This in situ XRD study of Ti_3_AlC_2_ etching revealed the absence of MXene after electrochemical treatment in NH_4_Cl/TMAOH electrolyte. However, the formation of MXene was detected after etching with both 0.2 M and 25% (2.8 M) TMAOH solutions using a freshly ground Ti_3_AlC_2_ precursor. The interlayer distance of “pristine” MXene recorded in TMAOH solution was found to be significantly different (∼17.1Å) compared to that of water‐washed and dried MXene (∼15 Å) due to the effects of swelling. Incomplete conversion of the Ti_3_AlC_2_ into MXene is likely related to the accumulation of reaction products. Reproducibility issues previously reported for chemical etching with TMAOH are likely related to the surface passivation layer, which can be disrupted by grinding the precursor Ti_3_AlC_2_ powder. Moreover, MXene found in earlier published experiments with electrochemical treatment of Ti_3_AlC_2_ could possibly form primarily due to a chemical reaction with TMAOH during etching and subsequent “washing” with more concentrated TMAOH.

## Graphical abstract

MXene synthesis by etching Ti_3_AlC_2_ with tetramethylammonium hydroxide (TMAOH) is shown to be reproducible if the passivation layer on the surface of crystallites is broken by rigorous grinding of the precursor powder. Widely cited as electrochemical, etching of Ti_3_AlC_2_ in aqueous NH_4_Cl/TMAOH (0.2 M) electrolyte is likely to yield MXene mostly due to TMAOH present in electrolyte and in post‐synthesis “washing”, which acts as a chemical etching agent.

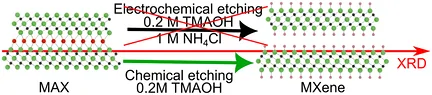

## 1 Introduction

MXenes are materials composed of 2D sheets of metal carbides terminated with a variety of functional groups [1, 2]. These materials have attracted a lot of attention over the last decade as promising for many applications, such as energy storage [3], sensors [4], membranes [5], and the removal of pollutants from wastewater [6]. The properties of MXenes are strongly dependent on the type of functional groups terminating the 2D sheets (e.g., fluorine, chlorine, hydroxyls, epoxy, etc), while the type of functionalization is mostly defined by the selection of the synthesis method [7, 8, 9, 10, 11].

Ti_3_C_2_T_x_ is the most extensively studied member of the MXenes family, synthesized by etching Al from the precursor Ti_3_AlC_2_ [7, 12]. The earliest reported method for etching Al was by using HF, thus providing MXene with 2D sheets terminated mainly with fluorine [12]. This method was later modified by using HF generated “in situ” by mixing LiF and HCl solutions [13]. Over the past decade, numerous alternative synthesis routes have been proposed to eliminate hazardous HF and to reduce environmental hazards. For example, etching of the MAX phase in molten salts is both fluorine‐free and water‐free [14, 15, 16, 17, 18, 19, 20]. The molten salt method yields fluorine‐free MXene, but it requires annealing at relatively high temperatures. Other fluorine‐free MXene synthesis methods include, for example, etching of precursor MAX phase with halogens (iodine or bromine) [21], NaOH [22, 23], and tetramethylammonium hydroxide (TMAOH) [24]. The etching with basic solutions was reported to face a major obstacle in the form of a surface oxide passivation layer, which was removed either by pre‐treatment with HF [24] or by using high‐temperature (270°C) hydrothermal treatment [23].

Electrochemical methods of Ti‐MXene synthesis are some of the most attractive alternatives as they are performed at ambient temperature [25] and can be modified by selecting a variety of electrolytes, including completely fluorine‐free ones [26, 27]. However, electrochemical etching of the MAX phase has proven challenging and does not consistently result in MXene formation [28].

One of the most frequently cited studies on the electrochemical synthesis of MXene reported the use of a binary aqueous electrolyte composed of 1 M NH_4_Cl and 0.2 M TMAOH [29]. According to the study, this method yields hydroxyl‐terminated Ti_3_C_2_. Notably, the key step of post‐synthesis treatment involved the “delamination” of Ti_3_C_2_T_x_ in a 25% (2.8 M) TMAOH solution [29]. However, etching of the MAX phase with TMAOH solutions alone has also been reported as a method for aluminium removal and subsequent MXene formation [24].

The use of TMAOH for post‐synthesis delamination of MXene has also been reported in several other studies as a routine component of delamination procedures [10, 30, 31]. Therefore, the role of TMAOH in the electrochemical etching of the MAX phase remains ambiguous, as does the mechanism of non‐electrochemical synthesis of MXene using TMAOH. It is worth noting that neither approach has been widely adopted for MXene synthesis. To the best of our knowledge, there is no independent verification of MXene synthesis via electrochemical etching of the MAX phase in NH_4_Cl/TMAOH electrolyte, despite the method being cited at the moment over 1000 times. Moreover, some subsequent studies have reported that TMAOH etching is not reproducible [10].

Therefore, we conducted an in situ XRD experiments using synchrotron radiation for a comparative study of the electrochemical etching of Ti_3_AlC_2_ (MAX phase) in an NH_4_Cl/TMAOH electrolyte, following the procedure described in Yang et al. [29], and a purely chemical etching method using TMAOH. In situ time‐resolved experiments enable direct characterization of the MXene structure during its formation directly in the etching solution, thus providing information about “pristine MXene” that has not been subjected to post‐synthesis washing treatments [32]. It is important to note that the structure of “pristine MXene” is intrinsic for the material formed directly in the etching solution and studied directly in the etching solution in a saturated swelling state [32, 33]. It is well known that swelling of MXene in aqueous solutions results in expansion of c‐lattice due to intercalation of water and ions into the inter‐layer spaces [13, 32, 33, 34, 35, 36, 37]. Moreover, swelling of MXene structure in liquid media is a common effect reported for many other polar solvents [35, 38]. The structure of pristine MXene transforms into a “standard” MXene after removal of etching solution and extensive washing [32, 33].

Our recent studies have provided valuable experience with in situ XRD as a powerful technique for investigating the electrochemical synthesis of 2D materials using miniature reaction cells [39]. Therefore, we designed a microscale electrochemical cell that enables experiments with the electrochemical etching of the MAX phase, as described in Yang et al. [29], while simultaneously recording time‐resolved XRD data to monitor the phase composition of the electrode material and elucidate the process of MXene formation.

In this study, no MXene was observed, even after prolonged electrochemical etching of the MAX phase in a 1 M NH_4_Cl/0.2 M TMAOH electrolyte. Following the exact procedure described in the earlier study (Yang et al. [29].) is not sufficient for producing MXene. Detailed investigation of possible parameters (not explicitly described in original report) which might result in electrochemical synthesis of MXene is out of scope of our study. Further experiments revealed that purely chemical etching with 0.2 and 2.8 M TMAOH solutions is sufficient to induce partial transformation of the MAX phase into MXene, provided the precursor material is finely ground to disrupt the surface passivation layer. Therefore, we have addressed a long‐standing controversy surrounding some of the most frequently cited methods for synthesizing MXenes. Our results suggest that TMAOH treatment, commonly used as part of post‐synthesis delamination protocols, is likely not limited to simple “washing”, but must be regarded as a step that facilitates the removal of residual aluminium from etched MAX phase materials.

## 2 Results and Discussion

**FIGURE 1:**
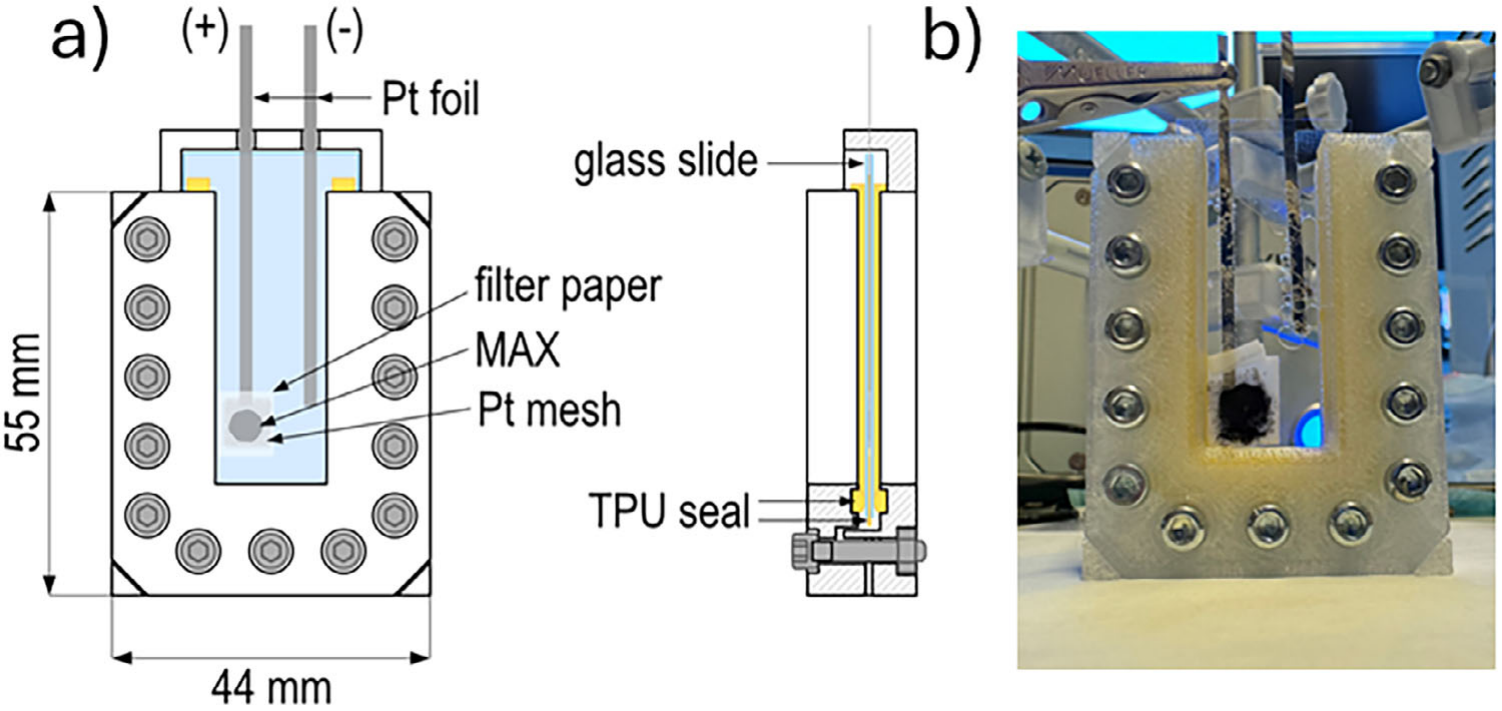
(a) Scheme of the micro electrochemical cell designed for in situ XRD experiments (see details in Supporting Information file). (b) Image of the cell in a process of operation.

The in situ XRD characterization of electrochemical MAX phase etching was performed using a specially designed microcell with two electrodes located between parallel glass plates, providing approximately 0.8 mm of space for the electrolyte. The MAX phase powder was attached to one of the electrodes using a platinum mesh (see experimental section for details) (Figure 1a). The potentiostatic curve recorded during in situ XRD investigations of electrochemical MAX phase oxidation is presented in Figure S1.

This cell is very similar to the cell used in the original report on electrochemical MXene synthesis [29], except for its significantly smaller size. The cell was mounted perpendicular to the incident X‐ray beam, as shown in Figure 1b. XRD data were first recorded from the MAX phase electrode in the empty cell, then in the cell filled with 1 M NH_4_Cl/0.2 M TMAOH electrolyte and continuously recorded during potentiostatic oxidation at a constant applied voltage of 5 V.

**FIGURE 2:**
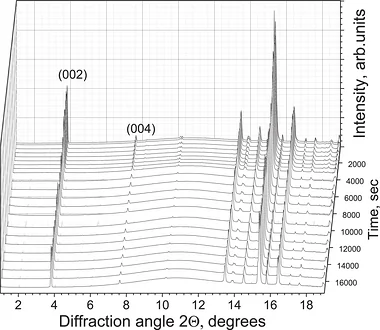
XRD patterns recorded in situ during potentiostatic oxidation of MAX phase in 1 M NH_4_Cl/0.2 M TMAOH electrolyte at constant applied voltage of 5 V. The structure of precursor Ti_3_AlC_2_ (MAX phase) remained unchanged even after prolonged treatment. λ=0.61992Å.

Surprisingly, MXene formation was not observed even after prolonged electrochemical treatment, and the structure of the precursor Ti_3_AlC_2_ remained unchanged (Figure 2). The XRD pattern of precursor MAX phase confirms the high purity of the precursor material (P6_3_/mmc space group with *a* = 3.071 Å and *c* = 18.577 Å) [12, 14]. Small impurities of Al_2_O_3_ and TiC have also been detected in the precursor powder. However, no additional reflections due to etching were detected even after prolonged electrochemical treatment. The experiment was repeated in an ex‐situ variant with a standard‐sized electrochemical cell. The data recorded before and after the laboratory scale electrochemical treatment of MAX phase at an applied voltage 5 V showed the same result with no MXene formation observed after 5 h of treatment (see Figure S2).

It needs to be noted that because of high voltage applied between the electrodes, different side electrochemical reactions will also occur on the current collector and working electrode surfaces, such as water electrolysis or chlorine formation. However, high voltage should also force simultaneous removal of aluminium from the MAX phase and MXene formation. This process can be described based on the reactions proposed in Yang et al. [29]:

The theoretical charge density needed for full transformation of MAX into MXene is equal to 2479 C g^−1^ (see Supporting Information file). Based on the laboratory scale electrochemical oxidation of MAX phase (Figures S3 and S4) the charge density delivered to the working electrode was 11 times larger. Therefore, it can be assumed that at least some part of MAX phase should be transformed into MXene during its electrochemical treatment in binary electrolyte. However, XRD data do not reveal any evidence for the formation of crystalline MXene phase. Additionally, SEM observations of electrochemically treated MAX phase in binary electrolyte do not reveal any significant changes in its surface morphology (Figure S5)

**FIGURE 3:**
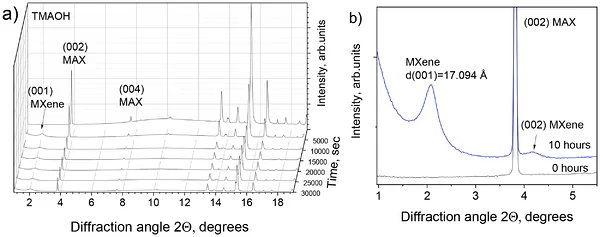
(a) XRD patterns recorded during ∼10 h of etching of the MAX phase with 25% TMAOH (every 5000^th^ pattern shown), (b) Low‐angle part of the first and last XRD patterns recorded during the etching. λ=0.61992Å.

It should be noted that the original study of electrochemical etching did not report XRD data for the synthesized material prior to TMAOH “washing” [29]. Therefore, we performed an additional in situ XRD experiment with the etching of Ti_3_AlC_2_ with 25% (2.8 M) TMAOH in order to verify if the post‐synthesis “washing” with this solution could result in the MXene formation (Figure 3).

**FIGURE 4:**
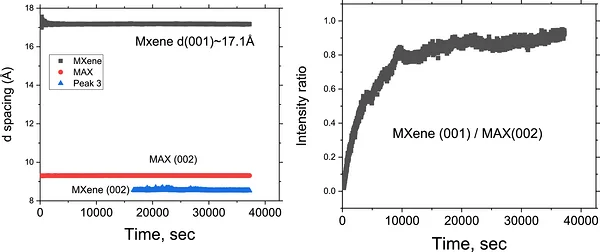
(a) Time evolution of (001) and (002) reflections of MXene and d(002) of MAX phase during TMAOH treatment at ambient temperature; (b) Evolution of intensity ratio for MXene (001) and MAX phase (002) reflections.

Analysis of the XRD data recorded during the etching shows that treatment with TMAOH results in partial conversion of MAX phase into MXene. The (001)‐reflection of MXene was first detected after ∼ 5 min of etching, the intensity of this reflection increased rapidly over ∼3 h, and then nearly stabilized. The d(001) of MXene remained unchanged over the whole period of etching at about 17.1 Å (Figure 4a). Note that the XRD patterns were recorded in these experiments from “pristine” MXene directly in the etching solution. Therefore, the d(001) value of 17.1Å corresponds to the structure expanded due to swelling in TMAOH solution and cannot be directly compared to the value of ∼15 Å reported in Xuan et al. [24] for water‐washed and dried MXene. The reaction rate slowed down after ∼3.5 h, as revealed by the time evolution of the intensity ratio of MXene (001) and MAX phase (002) reflections (Figure 4b).

A similar experiment was performed using a MAX phase precursor powder that had been more extensively ground in a mortar for approximately 10 min, in the hope that a smaller grain size would result in a faster and more complete reaction. The experiments were performed using a slightly different setup, with MAX phase powder enclosed in a plastic capillary and the etching solution added via a pipette (see our earlier work for details of this setup [32]). This experiment successfully reproduced the synthesis of MXene with d(001) ∼ 17.05 Å. Nevertheless, even after 9 h of etching, the reaction remained incomplete with a slightly higher intensity ratio for MXene (001) and MAX phase (002) reflections (∼1.3) as the only result of prolonged grinding of the precursor powder (Figures S6 and S7).

An additional in situ XRD experiment was performed using an etching solution composed of a mixture of TMAOH (20%) and NH_4_OH (∼16%). Spontaneous intercalation of NH_4_^+^ cations into the MXene lattice was reported in the Yang et al. [29]. However, adding NH_4_OH to the etching mixture did not result in additional c‐lattice expansion of MXene, indicating the absence of NH_4_^+^ cations intercalation. The MXene formed in this experiment still showed the same d(001) value of about 17.1 Å, while the rate of MAX phase to MXene conversion (estimated using intensity ratio I((001)MXene/(002)MAX)) remained similar to that of the experiment with ammonia‐free TMAOH etching.

Encouraged by the in situ XRD results, we attempted to synthesize a bulk amount of MXene using etching with TMAOH, but no MXene was formed in our first attempt, consistent with reports of irreproducible synthesis [10]. Puzzled by the difference between in situ and ex situ synthesis, we suggested that it might originate from the fine grinding applied to the MAX phase sample for in situ XRD experiments, as opposed to the nearly complete absence of grinding in experiments with bulk powder samples. Pre‐treatment with diluted HF was employed in the original report of MXene synthesis by TMAOH etching [24] to remove the “passivation” surface layer from the precursor MAX phase. Assuming that the passivation layer is indeed present in air‐stored MAX phase, using freshly ground powder might resolve the issue similarly to pre‐treatment with HF.

Therefore, we performed two additional ex situ experiments: one with MAX phase powder pre‐treated with a LiF + HCl solution for 30 min (without grinding), and another with the powder carefully crushed in a mortar for approximately 10 min. Both samples were then treated with 0.2 M TMAOH (as in electrochemical etching reported in Yang et al. [29]) for 24 h with stirring applied.

**FIGURE 5:**
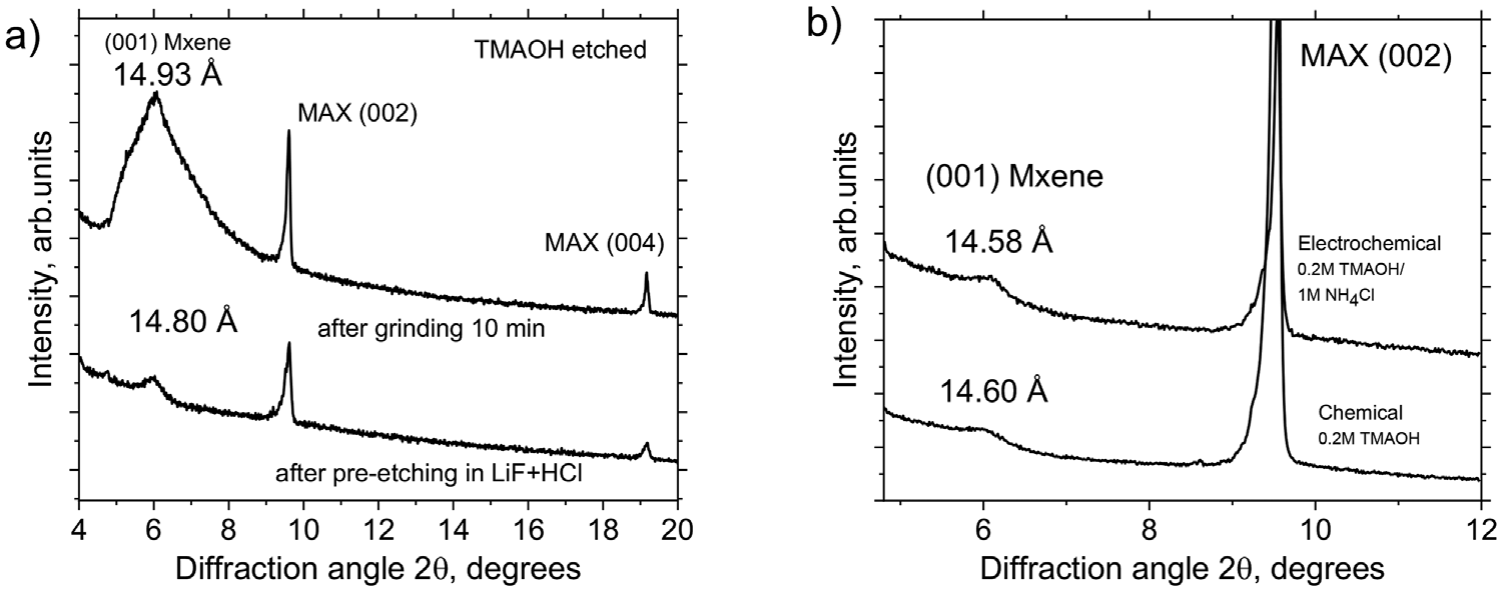
XRD patterns recorded from a sample of MAX phase: (a) TMAOH (0.2 M) etched for 24 h after pre‐treatment with HCl + LiF for 30 min, and from the sample vigorously crushed in a mortar for 10 min and then etched with TMAOH (0.2 M) for 24 h. (b) Sample of MAX phase electrochemically etched in 0.2 M TMAOH/ 1 M NH_4_Cl (5 h) using MAX phase powder after 10 min of grinding and lightly ground MAX phase (5 min) etched in TMAOH (0.2 M). The patterns are scaled along the vertical axis for clarity. Cu Kα radiation.

Both samples showed partial transformation into MXene. Remarkably, MXene formation was observed even in experiments with a low concentration (0.2 M) of TMAOH and in the absence of electrochemical treatment. Moreover, the freshly crushed sample of MAX phase showed significantly larger yield of MXene, as indicated by the relative intensities of the main reflections, but at the expense of lower structural ordering, evident from the broad and asymmetric shape of the (001) MXene reflection (Figure 5a). Lighter grinding (5 min) resulted in normal, symmetrically shaped (001) reflection, but with a smaller yield of MXene after 24 h (0.2 M) TMAOH etching. Ball milling for 10 mins did not increase the yield of MXene, indicating that stronger grinding is not improving etching (Figure S8). Analysis of Al content was performed for ball milled sample using a solution of TMAOH remaining after etching. The ICP‐OES showed four order magnitude increase of Al content relative to the initial background value. Also, XRF analysis showed a change of Ti:Al ratio from 2.82 in the precursor to 3.49 after etching, thus confirming partial removal of Al with formation of MXene evidenced by XRD. The slight excess of Al in the MAX phase is due to Al_2_O_3_ impurity commonly found in commercial precursors and detected as a minor phase using XRD. Composition of this sample after etching is MAX:MXene was estimated using XRF as 80%:20% (molar).

The values of d(001) found in these two samples (14.8 Å and 14.9 Å) are in good agreement with d(001) ∼15 Å reported in the original report of MXene produced by etching MAX phase with a significantly more concentrated (25% or 2.8 M) TMAOH solution.

On the next step, we verified if the grinding of the MAX phase also assists in electrochemical etching. The precursor MAX phase was ground for 10 min followed by electrochemical etching in 1 M NH_4_Cl/0.2 M TMAOH for 5 h and water washing. A weak (001) reflection of MXene was found in this sample with a d‐spacing of 14.58 Å. A nearly identical d(001) = 14.60 Å was found in the sample after 5 min of grinding and 24 h of purely chemical etching in 0.2 M TMAOH (Figure 5b). Notably, the electrochemical etching reported in the Yang et al. [29] resulted in partial etching and much smaller interlayer distance of ∼11.3Å after delamination and re‐stacking.

Analysis of data presented above shows that the MXene observed in experiments with electrochemical treatments is likely produced by purely chemical TMAOH etching of Ti_3_AlC_2_ [29].

We were unable to find any other independent reports about the structure of MXene formed after electrochemical etching with NH_4_Cl and TMAOH. To the best of our knowledge, the only other study in which this etching method was tested showed no evidence of MXene formation [40]. The XRD reflection assigned in this study to MXene was shifted relative to the (002) of precursor Ti_3_AlC_2_ only by approximately 0.1 Å and likely originated from a slightly disordered precursor MAX phase. Moreover, the (002) reflection of MAX phase in this study was weak (barely recognizable in the background noise), indicating some issues with precursor materials [40]. While we cannot completely rule out that absence of electrochemical etching in our experiments is related to different properties of precursor MAX phase, electrochemical synthesis of MXene phase described in the Yang et al. [29] remains to be not confirmed independently. The MAX phase precursor used in this study was successfully etched to produce MXene by three other methods, including etching with HCl + LiF solution [32], molten salt etching [18] and NH_4_F + acetic acid etching [33].

The expanded c‐lattice of MXene, prepared using TMAOH treatment, was explained in earlier studies by the intercalation of Al(OH)_4_^−^ anions, forming additional layers on the surface of Ti_3_C_2_ sheets [24]. We are unable to verify Al(OH)_4_^−^ termination of titanium carbide sheets in our experiments, as the MXene phase formed in our experiments was strongly disordered, and only partial etching was achieved. Moreover, another study involving basic etching of MAX phase (with NaOH at elevated conditions [23]) suggested that water‐soluble Al(OH)_4_^−^ should not form under conditions of confinement in the MXene lattice, giving priority to water‐insoluble Al hydroxides (Al(OH)_3_), and dehydrated aluminium oxide hydroxide AlO(OH). It seems possible that these reaction products formed during the etching process are accumulating in the MXene interlayers, preventing the complete conversion of the MAX phase into MXene.

**FIGURE 6:**
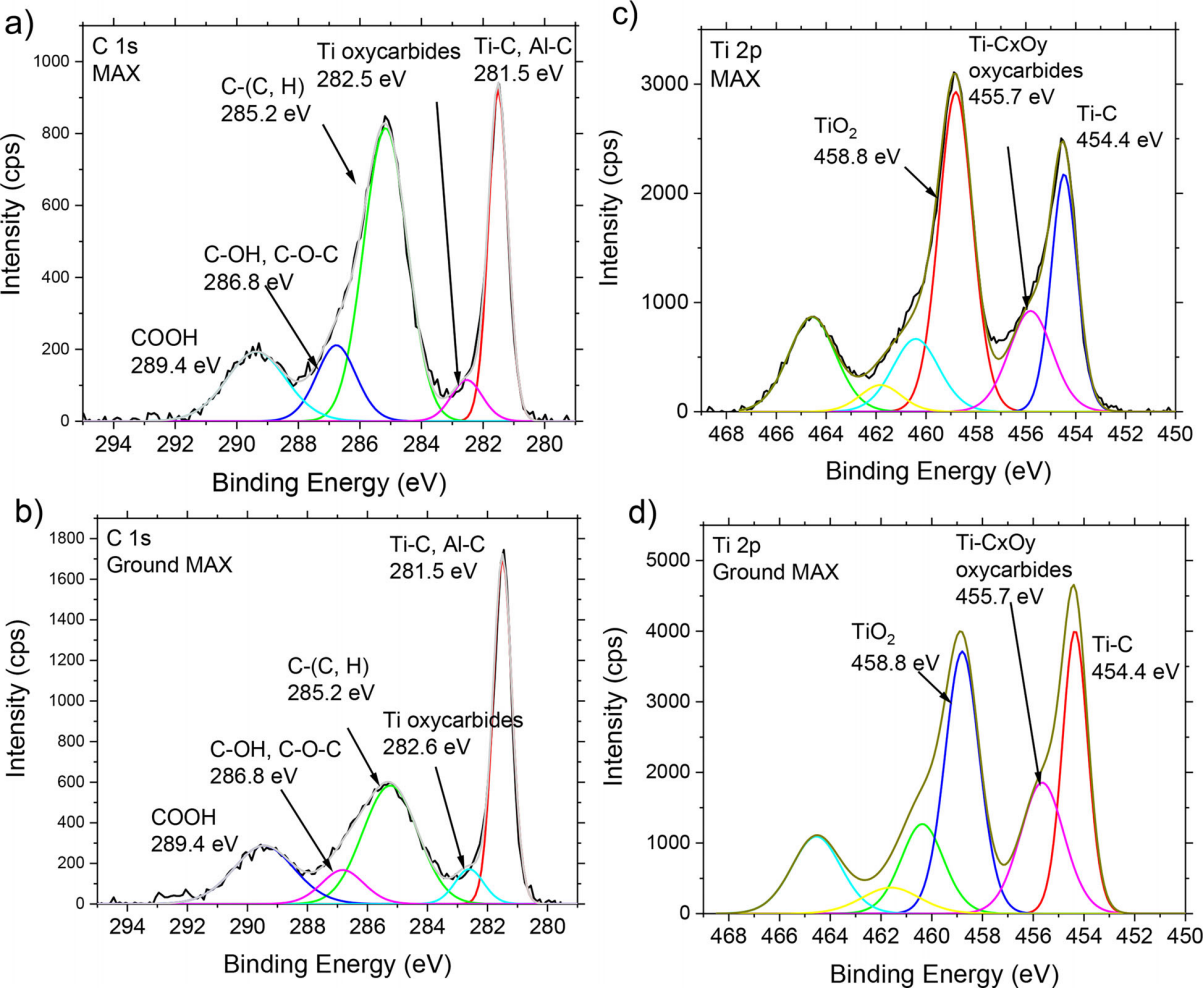
Ti 2p and C1s XPS spectra recorded from precursor (air stored) Ti_3_AlC_2_ sample before and after grinding: C 1s (a and b) and Ti 2p (c and d).

It can be concluded that our experiments confirm the existence of a passivation layer on the surface of air‐stored MAX phase. This passivation layer can be broken using simple grinding of the MAX phase precursor. This effect was verified by comparing XPS spectra of Ti_3_AlC_2_ before and immediately after crushing (Figure 6). Unlike XRD, which is sensitive only to crystalline phases, XPS is a surface‐sensitive method that allows for the evaluation of the composition of a very thin passivation layer. The spectra of ground MAX phase showed a smaller amount of TiO_2_ in Ti 2p (Figure 6c,d) and a smaller amount of carbon due to C─C (or C─H) in C 1s (Figure 6a,b). This result is expected if the thin passivation layer consists mainly of TiO_2_ and some amorphous carbon, possibly with hydrocarbons adsorbed on the surface of the crystallites. There was no difference in XPS spectra in the Al 2p part, indicating that the passivation layer is not related to aluminium oxide (Figure S9). The XPS data confirm that crushing helps to expose not passivated inner parts of MAX phase grains/crystallites, making it accessible for etching.

Obviously, grinding of the MAX phase powder is a much easier method to address surface passivation compared to the initially reported etching with an HF solution (Xuan et al. [24]). However, the reaction remains incomplete even in experiments with carefully ground powders.

The results presented above indicate that MXene formation in the electrochemical etching of MAX phase in 1 M NH_4_Cl/ 0.2 M TMAOH was likely due to experiments performed with a crushed sample of MAX phase. However, the MXene formation described in this study was likely to occur even without electrochemical treatment simply due to chemical etching in the electrolyte solution with 0.2 M TMAOH. Moreover, additional “washing” with a 25% TMAOH solution [29] possibly contributed to an even higher yield of MXene.

The main result of the in situ XRD experiments with TMAOH etching is the demonstration of “pristine” MXene interlayer distance of ∼17.1 Å, as recorded directly in TMAOH solution. This value is only about 2 Å larger than the value recorded from MXene after water washing. It should be noted here that swelling of MXene in water and aqueous solutions is very common effect reported in many studies, and the exact value of c‐lattice expansion in aqueous solution depends on many factors, including concentration and nature of dissolved ions or molecules [13, 32, 33, 34, 35]. The 17.1 Å value is also about 6 Å larger than the MXene reported in earlier electrochemical experiments (∼11.3 Å). It should be noted that the latter value is also similar to the inter‐layer distance of ∼12 Å reported in experiments with hydrothermal synthesis of MXene by NaOH etching [23]. Therefore, it can be suggested that the MXene c‐lattice formed after etching away Al is accessible to an aqueous solution of TMAOH, with etchant molecules capable of diffusing along the interlayers and expanding them. The etching reaction slows down after a few hours and remains incomplete. A relatively large part of the precursor MAX phase remained unreacted even after prolonged treatment, which is likely due to passivating oxide layers still present around some precursor crystallites. Alternatively, some incomplete removal of the reaction products (e.g., Al(OH)_3_ or AlO(OH) [23]) prevents diffusion of TMAOH into the deeper parts of interlayers, further away from the entrance points. In the latter case, the core of MAX phase crystallites will remain unreacted. Precipitation of Al(OH)_3_ occurs at lower pH values. Therefore, insufficient access of TMAOH into the deeper parts of MXene interlayers might result in local decrease of pH and precipitation of aluminium hydroxide. Revealing this contamination by XRD can be prevented by amorphous structure and extremely small sub nm width of MXene interlayers.

Finally, it is interesting to discuss some implications of TMAOH etching results presented above for optimization of MXene synthesis. Treatment with TMAOH is a part of standard post‐synthesis procedure aimed on delamination of MXene produced by acidic etching [10, 30, 31]. However, our results demonstrate that acidic and basic treatments can be complimentary for producing high quality 2D sheets of MXene. The mechanisms of acidic and basic etchings are fundamentally different: the TMAOH ions weaken the Ti‐Al bonds through hydrolysis, while the large cations simultaneously intercalate between the MAX phase layers. The Al removed form MAX phase by reaction with TMAOH is likely to form soluble aluminate complex, the tetrahydroxoaluminate ion Al(OH)_4_^−^. HF acts as a strong corrosive agent that removes Al layers by forming soluble AlF_x_. Results presented here allow us to suggest that TMAOH treatment is likely to result in the removal of some residual Al atoms or reaction byproducts (e.g., Al(OH_3_) more easily etched by alkaline treatment rather than acidic. It is possible that relatively few Al atoms remaining between Ti_3_C_2_ layers after acidic treatment provide “anchoring” effects preventing delamination. Some interstitial Al not accessible for acid treatment and removed by the reaction with alkaline TMAOH could be feasible explanation of the observed delamination. Therefore, it is likely that TMAOH serves as an additional etching agent rather than simply assisting delamination. Using TMAOH as a primary etching agent is fluorine free method which provides mostly ‐OH and oxygen terminated MXene but incomplete MAX phase to MXene conversion hindrance needs to be addressed for more this method to become competitive in bulk production.

## 3 Conclusion

In summary, our study resolves the controversy surrounding MXene synthesis, achieved by etching the Ti_3_AlC_2_ phase with TMAOH [29] and electrochemical etching of the MAX phase in NH_4_Cl/TMAOH solutions [29].

– Our initial attempts to replicate the synthesis of MXene using these two methods were unsuccessful, most likely due to a passivation layer forming on the surface of air‐stored precursor crystallites.
– This layer can be cracked by vigorous grinding of the precursor powder sample, which exposes the inner parts of the MAX phase crystallites. TMAOH etching of MAX phase powder performed directly after mechanical activation resulted in successful etching and MXene formation.
– In situ XRD data recorded during etching showed d(001) = 17.1 Å for the “pristine” MXene immersed in the etching TMAOH solution. A smaller value of ∼15 Å was recorded for the interlayer distance of MXene after water washing and drying.
– Therefore, we provide independent confirmation of the results reported in the Xuan et al. [24] and not reproduced in some later studies [10]. We note that using freshly ground powder is a much easier method to achieve reproducible etching with TMAOH compared to HF pre‐etching, originally proposed for removing passivation layers from precursor MAX phases [24].
– Electrochemical etching in an NH_4_Cl/TMAOH solution (as in Yang et al. [29]) resulted in the formation of MXene in low yield using a freshly ground precursor MAX phase. However, this MXene is likely to originate from purely chemical etching with 0.2 M TMAOH present in the electrolyte.

Our results also demonstrate that post‐synthesis “washing” or “delamination” with 25% (2.8 M) TMAOH must be considered as a part of the Al etching procedure. TMAOH treatment used post‐synthesis (e.g., after LiF+HCl etching) is likely to assist in dispersing MXene sheets by removing traces of Al remaining in the material. Moreover, MXene formation was observed even in experiments with a diluted 0.2 M TMAOH solution, previously used in the electrochemical synthesis of MXene Yang et al. [29]. Therefore, it cannot be ruled out that some part of MXene formed after electrochemical treatment of Ti_3_AlC_2_, followed by “washing” with 2.8 M TMAOH [29], originated from purely chemical etching of Al from the MAX phase. However, significant difference is found in the values of d(001) found after TMAOH etching (∼15 Å) and d(001) of MXene reported in the Yang et al. [29].(11.3 Å).

It cannot be ruled out that using a differently prepared or stored MAX phase precursor could still be the reason for the absence of electrochemical etching in the NH_4_Cl/TMAOH electrolyte in our experiments. However, it should be noted that to the best of our knowledge, there is no independent confirmation of electrochemical MXene synthesis using this method [29].

Moreover, the existence of passivation layers on the surface of powder grains of air‐stored MAX phase, confirmed here, is likely to affect at least some other MXene synthesis methods and needs to be taken into account. We hope that the results presented here can help resolve the controversy surrounding both fluorine‐free MXene synthesis methods, which involve TMAOH either as the primary etching agent or as part of the electrolyte in the electrochemical method, thereby improving reproducibility in future studies.

## Conflicts of Interest

The authors declare no conflicts of interest.

## Supporting information




**Supporting File**: smtd70878‐sup‐0001‐SuppMat.docx.

## Data Availability

The data that support the findings of this study are available from the corresponding author upon reasonable request.
