# Supplementary material for: Addressing Reproducibility of MXene Synthesis Using Electrochemical and Chemical Treatment of Ti3AlC2 With Tetramethylammonium Hydroxide
Source: Small Methods. 2026 Jul 28;10(16):e70878. doi: 10.1002/smtd.70878 (PMC13505648; doi:10.1002/smtd.70878)
Supplement: Supplementary file 1 [file SMTD-10-e70878-s001.docx]

Supporting information file

**Addressing reproducibility of MXene synthesis using electrochemical and chemical treatment of Ti_3_AlC_2_ with tetramethylammonium hydroxide**

Bartosz Gurzęda,^1^ Nicolas Boulanger,^1^ Mads Ry Vogel Jørgensen^2,3^, Innokenty Kantor,^3^ Christoph Hennig,^4^ Alexandr V. Talyzin*^1^

*^1^Department of Physics, Umeå University, Umeå, SE-901 87, Sweden. E-mail: alexandr.talyzin@umu.se*

*^2^ Department of Chemistry and iNANO, Aarhus University, Langelandsgade 140, DK-8000, Aarhus C, Denmark.*

*^3^ MAX IV Laboratory, Lund University, Fotongatan 2, 22594, Lund, Sweden.*

*^4^European Synchrotron Radiation Facility, The Rossendorf Beamline Grenoble 38043, France.*

1. **Experimental details**

**Characterization methods**

The ex situ XRD characterization of materials was performed using a Panalytical X'pert X-ray diffractometer with Cu Kα radiation in Bragg-Brentano geometry. XPS spectra were recorded using a Kratos Axis Ultra electron spectrometer equipped with a delay line detector. A monochromatic 150 W Al Kα source was used as the excitation source. A hybrid lens system with a magnetic lens, providing an analysis area of 0.3 × 0.7 mm, and a charge neutralizer was used for the measurements. The binding energy scale was adjusted with respect to the C1s line of aliphatic carbon, set at 285.0 eV. Survey spectra were recorded to evaluate the element composition of samples, and high-resolution spectra were recorded for the main elements. All spectra were processed with the Kratos software.

The main part of the in situ XRD experiments was recorded at the DanMAX beamline at the MAX IV synchrotron radiation facility. The diffraction data (10 frames per 1 s) were collected using radiation wavelength of 0.6199 Å (20.00 keV) on a DECTRIS PILATUS3 X 2M CdTe area detector. The wavelength and geometry of the instrument were refined using a LaB_6_ standard (NIST SRM660c) using the program PyFAI.^[1]^ The raw area detector data were azimuthally integrated to intensity versus 2θ using the MatFRAIA algorithm.^[2]^

Part of the in situ XRD data were collected at the Rossendorf beamline BM20 at ESRF, using an X-ray wavelength of λ = 0.72769 Å (17038 eV). The beam diameter was 100 μm. Experimental data were collected in transmission geometry using a DECTRIS PILATUS3 X 2M Si area detector and extracted with the Bubble software. The detector geometry parameters were calibrated with PyFAI using the NIST LaB6 standard reference material.

**Chemicals**

Precursor MAX phase Ti_3_AlC_2_ was purchased from Sigma Aldrich (≥99.99%) and characterized by XRD, XPS, and Raman spectroscopy, confirming the expected properties of the material. Tetramethylammonium hydroxide pentahydrate (98% purity) was purchased from Thermo Scientific.

2. **Electrochemical etching of MAX phase using microscopic cell designed for in situ XRD experiments**

In-situ electrochemical etching of Ti_3_AlC_2_ was studied using synchrotron XRD in a specially designed 3D printed electrochemical reactor (Fig. 1 in the main text). The reactor was made of a PETG body holding two glass slides (50 x 24 x 0.2 mm) separated by 3D printed TPU seals. The distance between the glass slides was equal to 0.8 mm. The working electrode, composed of MAX powder, was sandwiched between a Pt mesh (7 x 7 mm) and two filter paper pieces (9 x 9 mm) and remained in contact with a Pt foil (60 x 2 mm). Pt mesh as well as Pt foil was playing the role of a current collector. Note that during the electrochemical MAX oxidation, numerous oxygen bubbles formed on the working electrode. The design of the reactor allowed for constant contact between the MAX powder and both the electrolyte and the current collector. Pt foil was also used as a counter electrode. A binary aqueous solution consisting of 1 M ammonium chloride (NH_4_Cl) and 0.2 M tetramethylammonium hydroxide (TMAOH) was used as the electrolyte. MAX powder was anodically oxidized with a constant voltage of +5 V for 5 hours.

We note that a detailed characterization of the precursor MAX phase was performed.

3. **Laboratory scale electrochemical etching of MAX phase**

Electrochemical etching of Ti_3_AlC_2_ was additionally performed on a laboratory scale. For this purpose, 200 mg of MAX powder was placed between two Pt meshes (18 x 14 mm). The Pt mesh with MAX powder was then put between two filter paper pieces (20 x 18 mm) and held together by polytetrafluoroethylene (PTFE) frames. The Pt foil connected to the Pt mesh served as a current collector. The electrode was immersed in ~80 mL of a binary electrolyte consisting of 1 M ammonium chloride (NH_4_Cl) and 0.2 M tetramethylammonium (TMAOH) hydroxide. Pt foil was playing the role of the counter electrode. The MAX powder was anodically oxidized at a constant voltage of +5 V for 5 hours, and the electrolyte was continuously stirred magnetically. After the process, electrochemically oxidized Ti_3_AlC_2_ was washed out from the Pt mesh and paper filter using DI water and washed of the residues using a vacuum filtration system.

**Figure1S**. The galvanostatic curve recorded for potentiostatic oxidation of MAX phase in 1 M NH_4_Cl/0.2 M TMAOH electrolyte at constant applied voltage of 5 during in situ synchrotron radiation XRD measurements in capillary sized reactor.

**Figure 2S.** XRD patterns recorded from precursor MAX phase powder prior to etching, after immersing it in 1M NH_4_Cl/0.2 M TMAOH electrolyte for 5 hours, and after laboratory scale electrochemical treatment in this electrolyte for 5 hours. No MXene formation was found in this experiment. Some changes in the relative intensity of the main Ti_3_AlC_2_ reflections can be observed as a result of electrochemical treatment, but no new reflections due to MXene formation. The decrease of relative intensity for (00l) reflections indicates some disorder in the c-lattice introduced by the treatment. Cu Kα radiation.

**Figure 3S**. The galvanostatic curve recorded during the laboratory scale electrochemical oxidation of MAX phase in 1 M NH_4_Cl/0.2 M TMAOH electrolyte at constant applied voltage of 5 V.

**Figure 4S**. Electrochemical etching of MAX phase using laboratory scale synthesis setup.

4. **Theoretical charge density calculation for electrochemical etching of aluminium from MAX phase.**

The charge density needed for the removal of aluminium from MAX phase during the electrochemical oxidation in 1 M NH_4_Cl/0.2 M TMAOH electrolyte can be calculated based on theoretical reaction:

Ti_3_AlC_2_ + 2OH^-^ + 3Cl- → Ti_3_C_2_(OH)_2_ + AlCl_3_ + 5e^-^ (1)

and the formula:

Q = nF/M_Ti3AlC2_

were n being a number of the electrons involved in the reaction, F is Faraday constant (96485.33 C/mol), and M_Ti3AlC2_ [g/mol] is a molar mas of MAX phase. The calculated theoretical charge density for Al removal is equal to 2479.06 C/g, while the total charge delivered to the working electrode during the laboratory scale electrochemical oxidation of MAX phase was equal to ~5500 C. Because the mass of MAX phase used for the laboratory scale electrochemical oxidation was equal to 200 mg, the total charge density delivered to the working electrode was equal to ~27500 C/g and it was 11 times bigger than the theoretical charge density needed for full MAX phase transformation into MXene.

**Figure 5S**. SEM images recorded from precursor MAX phase (a,b), sample of MAX phase subjected to electrochemical etching treatment (c,d) and sample of MAX phase after partial conversion into MXene using TMAOH etching.

**Figure 6S**. XRD patterns recorded during the initial part (~6000 sec) of the etching treatment of ground Ti_3_AlC_2_ powder with 25% TMAOH. Further treatment resulted only in insignificant changes of relative intensity for (001) of MXene and (002) of MAX phase (λ=0.72769 Å).

**Figure 7S.** “Heat map” plot representing low-angle part of the XRD data recorded during etching of the MAX phase with 25% TMAOH over the full length of treatment (the same experiment as in Figure 3S (λ=0.72769 Å).

**Figure 8S.** XRD pattern recorded from sample of ball milled Ti_3_AlC_2_ after etching with TMAOH using the same procedure as the sample shown in Figure 5a. Cu Kα radiation.

**Figure 9S.** Al 2p XPS spectra recorded from air stored precursor Ti_3_AlC_2_ sample before and after grinding

References

[1]	J. Kieffer, V. Valls, N. Blanc, C. Hennig, *J Synchrotron Radiat* **2020**, *27*, 558-566.

[2]	A. B. Jensen, T. E. K. Christensen, C. Weninger, H. Birkedal, *J Synchrotron Radiat* **2022**, *29*, 1-9.
